# Clinical Phase I Study of TAS102/Irinotecan/Bevacizumab Combination Therapy in Japanese Patients With Unresectable Metastatic Colorectal Cancer (mCRC)

**DOI:** 10.7759/cureus.50431

**Published:** 2023-12-13

**Authors:** Tomohiro Adachi, Manabu Shimomura, Hiroyuki Egi, Wataru Shimizu, Yuji Takakura, Shoichiro Mukai, Masatoshi Kochi, Masanori Yoshimitsu, Takao Hinoi, Hideki Ohdan

**Affiliations:** 1 Gastroenterological and Transplant Surgery, Graduate School of Biomedical and Health Sciences, Hiroshima University, Hiroshima, JPN; 2 Gastroenterological Surgery, Hiroshima City North Medical Center Asa Citizens Hospital, Hiroshima, JPN; 3 Surgery, Chuden Hospital, Hiroshima, JPN; 4 Surgery, Chugoku Rosai Hospital, Hiroshima, JPN; 5 Gastroenterological Surgery, Higashihiroshima Medical Center, Hiroshima, JPN; 6 Surgery, Hiroshima City Hiroshima Citizens Hospital, Hiroshima, JPN

**Keywords:** phase i study, bevacizumab, irinotecan, ftd/tpi, metastatic colorectal cancer

## Abstract

Background: In this phase I study, we aimed to examine the safety of a triple combination (TAS-102/irinotecan/bevacizumab) therapy in patients with previously treated metastatic colorectal cancer (mCRC).

Methods: In the TAS-102 dose-escalation phase, we determined dose-limiting toxicity (DLT), estimated the maximum tolerated dose (MTD), and determined the recommended dose (RD); in the expansion phase, we evaluated safety. The RD was administered in advance for 10 patients. The TAS-102 dose was increased to 25-35 mg/m^2^ and administered orally twice on days 1-5 and 8-12. Irinotecan (100 mg/m^2^) and bevacizumab (5 mg/m^2^) were administered on days 1 and 15 of the treatment, respectively.

Results: Fifteen patients were enrolled in dose-escalation Levels 1-3, and ten in the expansion phase. A 30 mg/m^2^ TAS-102 dose at Level 2 was administered to three patients, with one presenting grade 4 neutropenia. A 35 mg/m^2^ TAS-102 dose at Level 3 was administered to five patients, with three patients presenting grade 4 neutropenia and grade 3 DLTs. We added three patients at Level 2 and set the MTD at 30 mg/m^2^, with no DLTs. The RD was fixed at 25 mg/m^2^, with no DLTs (N = 10) or treatment-related deaths. One patient showed complete response at Level 2, four presented partial response, and eleven individuals maintained stable disease for over four months. The median progression-free survival duration was 7.6 months, while the median overall survival period was 16.9 months.

Conclusion: The TAS-102/irinotecan/bevacizumab combination therapy was safe, effective, and well-tolerated in patients previously treated with mCRC.

## Introduction

Colorectal cancer (CRC) is the third most common type of cancer and the second leading cause of cancer-related deaths worldwide [1,2]. Treatment strategies for patients with metastatic CRC (mCRC) include surgery, radiotherapy, radiochemotherapy, systemic chemotherapy, and combination therapy. Chemotherapy is based on fluoropyrimidines, such as 5-fluorouracil (5-FU) and capecitabine, with the addition of oxaliplatin and irinotecan or a combination of both, which is a standard first- or second-line regimen [1,3,4]. Furthermore, additional concomitant use of molecularly targeted drugs is the current standard of care. A vascular endothelial growth factor inhibitor (bevacizumab) is used as a base for molecularly targeted drugs, but an epidermal growth factor inhibitor (e.g., cetuximab or panitumumab) is used for tumors containing the wild-type RAS gene. A human epidermal growth factor receptor 2 (HER2)-targeted inhibitor is used to treat HER2-amplified mCRC [5], and immunotherapy is used to treat mCRC with high microsatellite instability [6]. Their effective use prolongs the median survival time of patients with mCRC [7,8].

The formulation TAS-102 (Taiho Pharmaceutical, Tokyo, Japan) acts as an oral anti-tumor agent and consists of trifluridine (FTD; α,α,α-trifluorothymidine) and tipiracil hydrochloride [TPI; thymidine phosphorylase inhibitor;5-chloro-6-(2-iminopyrrolidin1-yl) methyl-2,4(1H,3H)-pyrimidinedione hydrochloride]. Trifluridine is an active anti-tumor component of TAS-102. The direct incorporation of FTD into DNA strands is considered to contribute to its anti-tumor effects by causing DNA dysfunction [9], and the incorporation of TPI inhibits FTD degradation and maintains blood levels [10]. This mechanism of action is different from the mechanisms of other cytotoxic and targeted agents, and TAS-102 is expected to be effective against various tumors resistant to other drugs.

In an international phase 3 comparative trial of TAS-102 and placebo in patients with mCRC who had received at least two prior lines of therapy, TAS-102 prolonged overall survival (OS) compared to the placebo (hazard ratio, 0.68; 95% confidence interval, 0.58-0.81 (P < 0.001); median OS, 7.1 vs. 5.3 months, respectively). Moreover, grade 3 or higher AEs occurred in 49% of patients and were controllable [11]. Based on these results, the TAS-102 standard dose of 35 mg/m^2^ was approved for patients previously treated for mCRC.

In a study conducted in vivo using CRC cell lines, the combined use of TAS-102 with either irinotecan or bevacizumab showed significantly greater anti-tumor activity than using each agent individually. In particular, the concomitant use of irinotecan with TAS-102 enhanced the anti-tumor activity of TAS-102 [12,13]. Building on the findings of this study, the first phase I trial on the combination of TAS-102 and irinotecan showed its improved anti-tumor activity compared with that of either agent alone in patients with previously treated mCRC. However, a higher rate of grade 3 or higher hematologic toxicity was observed with the combination than with either agent alone, suggesting the need for dose adjustments of TAS-102 and irinotecan [14]. Considering the results of various clinical trials with fluoropyrimidines, bevacizumab, and TAS-102 are expected to have non-overlapping toxicities [15-18].

We designed a two-part phase Ib dose-escalation/expansion study to investigate the anti-tumor efficacy and safety of TAS-102 by increasing the TAS-102 dose to its lowest level in combination with bevacizumab. During the dose-escalation phase, our focus was on assessing the safety of TAS-102 when administered in conjunction with irinotecan and bevacizumab, aiming to determine the maximum tolerated dose (MTD). We extended our evaluation to include safety considerations and preliminary activity assessments in the expansion phase.

## Materials and methods

Study design

**Figure 1:**
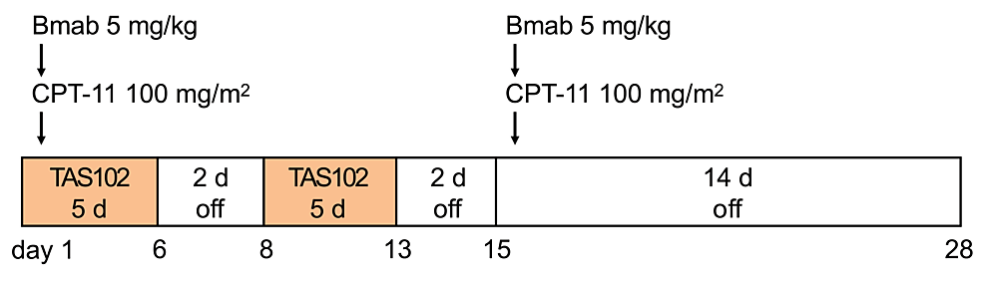
Study drug dosing schedule TAS-102 was administered orally twice on days 1–5 and 8–12. Irinotecan (100 mg/m^2^) and bevacizumab (5 mg/m^2^) were administered on days 1 and 15 of the treatment, respectively). Bmab- Bevacizumab; CPT-11- Irinotecan

This non-blind, single-arm, single-center study was conducted at Hiroshima University. This was a phase 1 trial that included a dose-escalation phase aimed at assessing safety and establishing the MTD of TAS-102 and irinotecan combined with bevacizumab in CRC treatment. This was followed by an expansion phase, exploring its safety. Participants were administered TAS-102 orally twice daily for the first 5 days of a 2-week treatment cycle, with bevacizumab administered intravenously on the first day of every cycle before irinotecan at the same time as TAS-102 (Figure 1).

Treatment was continued until disease progression as defined by Response Evaluation Criteria in Solid Tumors (RECIST) version 1.0 or clinical measures, intolerable adverse effects leading to more than three dosage reductions of TAS-102, request of a patient to end treatment, pregnancy, or decision of the physician to transition to a different cancer therapy.

**Figure 2:**
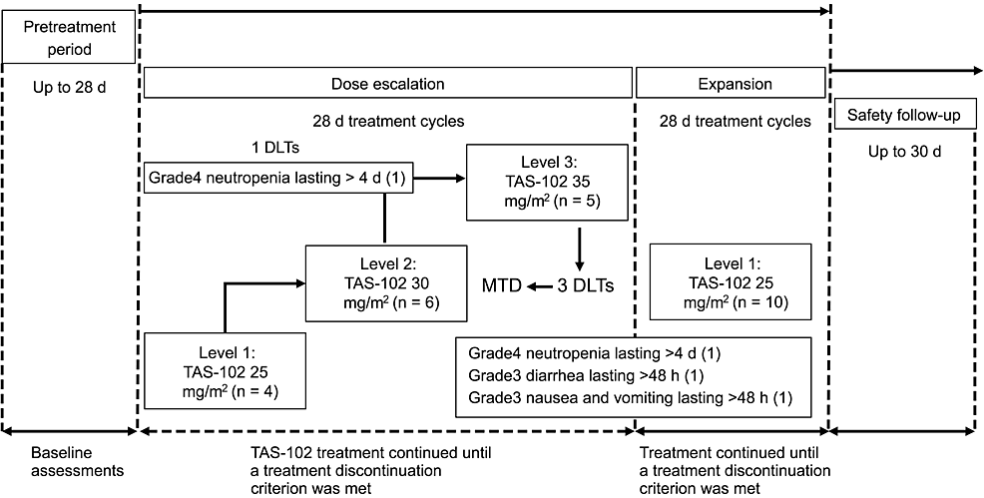
Study design of the dose-escalation and expansion phases. The dose escalation followed a 3+3 design to identify the maximum tolerated dose (MTD) of TAS-102 in patients with advanced colorectal tumors. In the dose escalation cohort, no DLTs were observed at Level 1. At Level 2, one DLT was observed in a patient who developed grade 4 neutropenia that persisted for over 4 days, and three DLTs were observed at Level 3. These findings led to establishing the MTD of Level 1 for the expansion phase. DLT: dose-limiting toxicity

The dose-escalation phase adhered to a classic 3 + 3 model (Figure 2), wherein patients in subsequent dose-level groups were administered increasing amounts of TAS-102 (25, 30, or 35 mg/m^2^/dose, twice daily).

At least three patients eligible for evaluation were treated at each level, and no less than six patients were treated at the MTD. Progression to the next dose level was only allowed after confirming that the preceding level was safe, per the protocol. Dose escalation between patients was not permitted. The MTD was identified as the highest dose at which <33% of the patients being evaluated experienced dose-limiting toxicity (DLT) during the first or second cycle of study drug administration. The expansion phase commenced after confirming the MTD, and only patients (mCRC) who received fluoropyrimidines, oxaliplatin, and bevacizumab were included.

The initial six patients were concurrently enrolled and administered the determined maximum tolerated dose (MTD) of TAS-102, along with irinotecan at 100 mg/m^2^ and bevacizumab at 5 mg/kg. The expansion phase involved conducting an interim safety assessment (ISA) to evaluate safety in the first six patients after two treatment cycles or treatment discontinuation, whichever came first. Further recruitment for the expansion cohort, which was set to receive the triple combination treatment (with a maximum of 10 patients), depended on the completion of the ISA and the absence of unexpected safety concerns. In the event of unforeseen adverse events (AEs), an additional safety review was scheduled for the subsequent six enrolled patients, and treatment was continued with necessary dose adjustments for bevacizumab.

Patient eligibility

The eligibility criteria were as follows: (1) Japanese individuals with pathologically verified inoperable metastatic or recurrent CRC who had previously shown disease progression after chemotherapy that included fluoropyrimidine and oxaliplatin; (2) the presence of at least one measurable lesion following RECIST 1.0; (3) no prior exposure to TAS-102 treatment; (4) age of 20-75 years; (5) Eastern Cooperative Oncology Group Performance Status (ECOG-PS) score of 0 or 1; (6) sufficient bone marrow function, including platelet count ≥ 75,000/mm3, hemoglobin level ≥ 9.0 g/dL, and neutrophil count = 1500/mm3; (7) acceptable liver function (bilirubin ≤ 1.5 mg/dL, aspartate and alanine aminotransferase levels ≤ 100 IU/L; and (8) sufficient kidney function (creatinine level ≤ 1.5 mg/dL). Individuals were not eligible if they (1) had hypersensitivity to bevacizumab, history of hemoptysis, contraindication for irinotecan, severe concurrent diseases, for example, active secondary cancer(s), nervous system metastasis, unmanageable infectious diseases, uncontrolled pleural effusion, ascites, or cardiac effusion, ileus, interstitial pneumonia, renal/liver failure or cerebrovascular disorders, uncontrolled diabetes or hypertension, uncontrolled diarrhea or jaundice, myocardial infarction, severe angina or New York Heart Association Class III or IV designation within 12 months prior to the registration, gastrointestinal bleeding, hepatitis B surface antigen-positive, hepatitis C or human immunodeficiency virus antibodies, immunosuppressive therapy requirement, uncontrolled mental illness or psychosis; (2) were undergoing certain treatments, including major surgery within 4 weeks prior to enrollment, wide field radiotherapy 4 weeks before enrollment, local radiotherapy 2 weeks before enrollment, use of other experimental drugs 4 weeks before enrollment, blood or granulocyte colony-stimulating factor (G-CSF) transfusion 14 days before enrollment, or severe kidney failure or proteinuria ≥2 + 7 days before enrollment; (3) had defecated ≥7 times/day or incontinence, thromboembolic event or severe pulmonary complaints within 6 months, incomplete wound healing or fracture, or predisposition to bleeding or antithrombotic medication; (4) were pregnant or lactating; or (5) had any reason deemed by the investigator making the patient unsuitable for participation. The study adhered to the Declaration of Helsinki and Japanese Good Clinical Practice guidelines. Written informed consent was obtained from all participants. The Ethics Committees of the participating institutions, including the Hiroshima University Hospital, approved this study (CRB6180006). This study was registered in the Japan Registry of Clinical Trials (jRCTs061180069).

Safety and efficacy assessments

The safety group consisted of individuals who were administered at least one dose of the experimental treatment. Safety evaluations were conducted from initial administration until a month after the final dose of the test drug. The AEs were classified following the Common Terminology Criteria for Adverse Events version 4.03 of the National Cancer Institute. Blood examinations and nonhematologic toxicity assessments were performed on days 1, 3, 5, 8, 10, 12, and 15-28. A DLT was characterized as an AE during the first or second cycle that met one of the following conditions: grade 4 neutropenia persisting over a week; feverish neutropenia, grade 4 thrombocytopenia, or bleeding accompanied by grade 3 thrombocytopenia; nonhematologic toxicity of grade 3 or toxicity; nausea, diarrhea, or vomiting of grade 3 or above persisting beyond 48 h; any toxic reaction linked to the study drug leading to over a 2-week treatment delay at the beginning of cycle 3; or any toxicity related to the study drug hindering the administration of 80% or more of the estimated cumulative dose during cycle 1 or 2.

We evaluated treatment response according to RECIST 1.1 criteria, conducting tumor assessments at the beginning and every four cycles (8 weeks) for the first 24 cycles or as medically necessary. Patients who had completed one or more cycles of the study medication and had undergone assessments for radiologic/clinical progression were deemed eligible for evaluation.

Statistical methods

Patient attributes and effectiveness and safety assessments were performed using statistical descriptions. The Kaplan-Meier approach was used to evaluate progression-free survival (PFS), and the Brookmeyer and Crowley technique was adopted to generate confidence intervals for the median PFS. All compilations and data were recorded using SPSS version 26 software provided by the IBM Institute (New York, NY, USA).

## Results

Participant demographics

The study involved 25 participants recruited between October 5, 2015, and December 16, 2022. Of these, 15 patients with CRC were split into three ascending dose groups, with 4-6 individuals per group. The remaining 10 participants were included in the expanded group (Figure 2). All participants received a minimum of one dose of the study drug. As of the June 30, 2023 data cutoff, 23 participants (23/25, 92%) had discontinued treatment because of disease progression. Furthermore, one patient (1/25, 4%) discontinued treatment because of non-hematological toxicity, and another patient (1/25, 4%) discontinued treatment because of undergoing radical surgery. The average age of the entire participant group (N = 25) was 64 (range 23-75) years, and the majority (22/25, 88%) of the patients had an ECOG-PS score of 0 (Table 1).

**Table 1:** Patient Characteristics EGFR: Epidermal growth factor receptor; KRAS: Kirsten rat sarcoma viral oncogene; ECOG: Eastern Cooperative Oncology Group The data has been represented as N, %, and median value (range).

	Level 1	Level 2	Level 3	Total
	(N = 14)	(N = 6)	(N = 5)	(N = 25)
	N (%)	N (%)	N (%)	N (%)
Median age (range), years	64 (23–72)	67(56–71)	55(23–75)	64 (23–75)
Sex				
Male	7 (50%)	3 (50%)	4 (75%)	14 (56%)
Female	7 (50%)	3 (50%)	1 (25%)	11 (44%)
ECOG Performance Status				
0	12 (85.7%)	5 (83.3%)	5 (100%)	22 (88%)
1	2 (14.3%)	1 (16.7%)	0 (0%)	3 (12%)
Diagnosis				
Recurrent	6 (42.9%)	3 (50%)	4 (80%)	13 (52%)
Unresectable	8 (57.1%)	3 (50%)	1 (20%)	12 (48%)
Primary Lesion				
Colon	8 (57.1%)	4 (66.7%)	4 (80%)	16 (64%)
Rectum	6 (42.9%)	2 (33.3%)	1 (20%)	9 (36%)
Number of metastatic sites				
1	3 (21.4%)	3 (50%)	3 (60%)	9 (36%)
2	8 (57.1%)	2 (33.3%)	2 (40%)	12 (48%)
3 or more	3 (21.4%)	1 (16.7%)	0 (0%)	4 (16%)
Histological Type				
Well-differentiated	2 (14.3%)	1 (16.7%)	2 (40%)	5 (20%)
Moderately differentiated	10 (71.4%)	5 (83.3%)	2 (40%)	17 (68%)
Mucinous	1 (7.1%)	0 (0%)	1 (20%)	2 (8%)
Unknown	1 (7.1%)	0 (0%)	0 (0%)	1 (4%)
Primary tumor resection	11 (78.6%)	5 (83.3%)	5 (100%)	21 (84%)
Adjuvant chemotherapy	4 (28.6%)	4 (66.7%)	4 (80%)	12 (48%)
Treatment line				
2nd line	3 (21.4%)	0 (0%)	4 (80%)	7 (28%)
3rd line	10 (71.4%)	5 (83.3%)	0 (0%)	15 (60%)
4th line	1 (7.1%)	1 (16.7%)	1 (20%)	3 (12%)
Prior treatment				
Oxaliplatin	14 (100%)	6 (100%)	5 (100%)	25 (100%)
Irinotecan	13 (92.3%)	5 (83.3%)	3 (60%)	21 (84%)
Bevacizumab	9 (64.3%)	6 (100%)	5 (100%)	20 (80%)
Anti-EGFR antibody	4 (28.6%)	1 (16.7%)	0 (0%)	5 (20%)
KRAS status				
Wild-type	7 (50%)	1 (16.7%)	2 (40%)	10 (40%)
Mutant	7 (50%)	5 (83.3%)	3 (60%)	15 (60%)
UGT1A1 polymorphism				
Wild-type	14 (100%)	6 (100%)	5 (100%)	25 (100%)

In total, 72% (18/25) of the patients had received three or more prior treatment regimens for advanced disease, and 84% (21/25) had previously received irinotecan. Additionally, 60% (15/25) of the patients harbored Kirsten rat sarcoma viral oncogene (KRAS) mutations. Details of RAS gene mutation subtypes were G12D in 6 cases, G12V in 4 cases, G12C in 1 case, G12S in 1 case, and G13D in 2 cases. All participants were identified as having the wild-type uridine diphosphate glucuronosyltransferase 1A1 (UGT1A1) gene.

Establishing the MTD

In the ascending dose cohort, no DLTs were observed at Level 1 (TAS-102, 25 mg/m^2^ twice daily). At Level 2 (TAS-102 30 mg/m^2^ twice daily), one DLT was observed in a patient who developed grade 4 neutropenia that persisted for over 4 days, coupled with febrile neutropenia. Three DLTs were observed at Level 3 (TAS-102, 35 mg/m^2^ twice daily). One patient developed grade 4 neutropenia lasting over 4 days, another developed grade 3 diarrhea and vomiting, and a third developed grade 3 reduced appetite and nausea. These findings led to establishing the MTD of TAS-102 25 mg/ m^2^ twice daily (Level 1) for the expansion phase.

Treatment safety

The median duration of treatment was 7.7 (ranging from 0.8 to 33.8) months in the overall patient population (N = 25) and 7.2 (ranging from 0.83 to 14.2) months in the expansion cohort. In Level 1, all patients received the intended target dose of TAS-102 at 100% (14/14). Each patient encountered one or more adverse events (AEs) of any origin, with 85.7% (12/14) of the patients experiencing AEs classified as grade 3. There were no deaths at Level 1. Commonly encountered treatment-related AEs are listed in Table 2.

**Table 2:** Treatment-related adverse events The data has been represented as N, %. AST: Aspartate aminotransferase; ALT: alanine aminotransferase

	Level 1 (N = 14)		Level 2 (N = 6)		Level 3 (N = 5)		Total (N = 25)	
	All grades	Grade 3/4	All grades	Grade 3/4	All grades	Grade 3/4	All grades	Grade 3/4
	N (%)	N (%)	N (%)	N (%)	N (%)	N (%)	N (%)	N (%)
Hematological toxicities	12 (85.7%)	12 (85.7%)	6 (100%)	4 (66.7%)	5 (100%)	4 (80%)	23 (92%)	20 (80%)
Neutrophil count decreased	12 (85.7%)	12 (85.7%)	6 (100%)	4 (66.7%)	5 (100%)	4 (80%)	23 (92%)	20 (80%)
White blood cell count decreased	12 (85.7%)	12 (85.7%)	6 (100%)	3 (50%)	4 (80%)	4 (80%)	22 (88%)	19 (76%)
Hemoglobin decreased	10 (71.4%)	1 (7.1%)	5 (83.3%)	1 (16.7%)	4 (80%)	0 (0%)	19 (76%)	2 (8%)
Platelet count decreased	3 (21.4%)	0 (0%)	3 (50%)	1 (16.7%)	2 (40%)	0 (0%)	8 (32%)	1 (4%)
Febrile neutropenia	1 (7.1%)	1 (7.1%)	1 (16.7%)	1 (16.7%)	1 (20%)	1 (20%)	3 (12%)	3 (12%)
Anemia	10 (71.4%)	1 (7.1%)	5 (83.3%)	1 (16.7%)	4 (80%)	0 (0%)	19 (76%)	2 (8%)
AST elevation	6 (42.9%)	1 (7.1%)	2 (33.3%)	0 (0%)	2 (40%)	0 (0%)	10 (40%)	1 (4%)
ALT elevation	6 (42.9%)	2 (14.3%)	3 (50%)	0 (0%)	2 (40%)	0 (0%)	9 (36%)	2 (8%)
Blood Albumin decreased	0 (0%)	0 (0%)	0 (0%)	0 (0%)	0 (0%)	0 (0%)	0 (0%)	0 (0%)
Protein total decreased	0 (0%)	0 (0%)	0 (0%)	0 (0%)	0 (0%)	0 (0%)	0 (0%)	0 (0%)
Non-hematological toxicities	11 (78.6%)	2 (14.3%)	6 (100%)	0 (0%)	5 (100%)	2 (40%)	22 (88%)	4 (16%)
Decreased appetite	9 (64.3%)	0 (0%)	6 (100%)	0 (0%)	4 (80%)	1 (20%)	19 (76%)	1 (4%)
Diarrhea	9 (64.3%)	0 (0%)	5 (83.3%)	0 (0%)	4 (80%)	1 (20%)	18 (72%)	1 (4%)
Stomatitis	3 (21.4%)	0 (0%)	3 (50%)	0 (0%)	3 (60%)	0 (0%)	9 (36%)	0 (0%)
Malaise	11 (78.6%)	1 (7.1%)	5 (83.3%)	0 (0%)	4 (80%)	0 (0%)	20 (80%)	1 (4%)
Nausea	7 (50%)	1 (7.1%)	3 (50%)	0 (0%)	4 (80%)	1 (20%)	14 (56%)	2 (8%)
Alopecia	2 (14.3%)	0 (0%)	0 (0%)	0 (0%)	0 (0%)	0 (0%)	2 (8%)	0 (0%)
Vomiting	2 (14.3％)	1 (7.1％)	2 (33.3％)	0 (0％)	2 (40％)	1 (20％)	6 (24％)	1 (4％)
Mucositis	2 (14.3)	0 (0％)	0 (0％)	0 (0％)	0 (0％)	0 (0％)	2 (8％)	0 (0％)
Proteinuria	1 (7.1%)	0 (0％)	0 (0％)	0 (0％)	0 (0％)	0 (0％)	1 (4%)	0 (0％)
Thrombosis	1 (7.1％)	1 (7.1%)	0 (0％)	0 (0％)	0 (0％)	0 (0％)	1 (4％)	1 (4％)

The most frequent treatment-related AEs were bone marrow suppression, malaise, decreased appetite, diarrhea, and nausea. Although nearly all symptomatic treatment-related AEs, including gastrointestinal symptoms, except vomiting, nausea, and malaise, were grade 2 or lower, grade 3 or higher treatment-related AEs were associated with bone marrow suppression. Six instances of grade 4 neutropenia occurred in 2 patients at Level 1, 2 at Level 2, and 2 at Level 3. Bone marrow suppression was reversible in all patients. None of the patients died within 90 days of treatment initiation or 30 days after treatment completion (discontinuation). None of the patients discontinued the study because of treatment-related AEs. One severe treatment-related AE (febrile neutropenia) occurred in one patient at Level 2 and one patient at Level 3, and it resolved with appropriate treatment. No deaths were reported in the expansion cohort at the data cutoff date. Collectively, no grade 5 treatment-related AEs were reported. This clinical trial demonstrated high hematotoxicity.

Treatment efficacy

Table 3 depicts a response rate of 14.3% (2/14) at Level 1, 50% (3/6) at Level 2, 0% (0/5) at Level 3, and an overall 20.0% (5/25) (with response durations of 9.3 and 33.8 months). At Level 2, a complete response rate of 16.7% (1/6) was observed. The disease control rates were 64.3% (9/14) at Level 1, 50% (3/6) at Level 2, 75% (4/5) at Level 3, and 64.0% (16/25) overall. The median PFS was 7.6 months, whereas the median OS was 16.9 months.

**Table 3:** Efficacy summary CR: complete response; PR: partial response; SD: stable disease; PD: progressive disease, NE: not evaluable The data has been represented as N, %.

	Level 1	Level 2	Level 3	Total
	(N = 14)	(N = 6)	(N = 5)	(N = 25)
	N (%)	N (%)	N (%)	N (%)
CR	0 (0%)	1 (16.7%)	0 (0%)	1 (4%)
PR	2 (14.3%)	2 (33.3%)	0 (0%)	4 (16%)
SD	7 (50%)	0 (0.0%)	4 (75%)	11 (44%)
PD	5 (35.7%)	3 (50%)	0 (0.0%)	8 (32%)
NE	0 (0%)	0 (0%)	1 (25%)	1 (4%)
Response Rate (CR + PR)	2 (14.3%)	3 (50%)	0 (0%)	5 (20%)
Disease Control Rate (CR + PR + SD)	9 (64.3%)	3 (50%)	4 (75%)	16 (64%)

## Discussion

Our phase I trial demonstrated the tolerability and initial efficacy of TAS-102 in combination with irinotecan and bevacizumab among patients previously treated for metastatic colorectal cancer (mCRC). During the dose-escalation phase, we observed four instances of dose-limiting toxicities (DLTs), including grade 3 fatigue and nausea, reduced appetite, and grade 4 neutropenia. Consequently, the Maximum Tolerated Dose (MTD) for previously treated mCRC patients was established as TAS-102 25 mg/m^2^ (administered twice daily on days 1-5 of a 14-day cycle), along with irinotecan 100 mg/m^2^ and bevacizumab 5 mg/kg (administered on day 1 of the 14-day cycle). In the subsequent expansion phase, the safety profile of the three-drug combination regarding the types and frequencies of adverse events (AEs) was comparable to that observed in Level 1. The most common AEs of any cause and grade included neutropenia, fatigue, nausea, and vomiting, while the most common grade ≥3 AEs were hematological, such as decreased white blood cell and neutrophil counts. However, effective management of AEs was achieved through dose adjustments, including delays and interruptions. No treatment-related AEs led to treatment discontinuation or death. These results validate the consistent safety profile of individual agents that posed no unexpected safety concerns.

Combining TAS-102 with other cytotoxic agents showed synergistic effects, whereas combining TAS-102 with irinotecan showed the most promising anti-tumor effects. Notably, SN-38, an active metabolite of irinotecan, induces DNA strand breaks and promotes G2/M arrest in combination with FTD [19]. The TAS-102 formulation has also been effective against human tumor cell lines resistant to 5-FU [20]. Hence, the TAS-102 and irinotecan combination is an innovative candidate for mCRC resistant to 5-FU-based initial chemotherapy. However, severe adverse effects have hindered its clinical application.

The MTD and dosing schedule of TAS-102 utilized in this study, which differs from the approved monotherapy dose and schedule, were chosen based on insights from prior preclinical studies and a phase I study conducted in Japan [12-14]. However, the standard FTD/TPI dosing schedule led to delayed myelotoxicity. Despite the initial anti-tumor effectiveness, all patients encountered grade ≥3 hematologic adverse events [14]. Through a strategy involving a reduction in irinotecan dosage and an increase in TAS-102 dosage, we not only improved the efficacy of TAS-102 but also observed lower frequencies of grade ≥3 hematologic adverse events at the recommended dose, thereby validating the current dosing schedule.

Most patients did not experience neutrophil recovery by day 28, prompting initiation of the next course of treatment after 35 days. We administered TAS-102 twice daily over a 35-day cycle. Despite the possibility of administering higher dosages as in other studies, it was not feasible in this trial [14,21]. The MTD may have been possible at Level 2; however, grade ≥3 neutropenia was observed in highly effective cases; therefore, dose reduction was required in the subsequent cycles, and the result was Level 1 dosage. Grade 3 neutrophil loss was virtually inevitable if the count was <1500 on day 15. Predicting future trends in neutrophil count reduction from the images in Fig 3 is vital. However, G-CSF administration is not mandatory, and recovery occurs naturally. Level 3 demonstrated greater non-hematological toxicity than Levels 2 and 1. This study also demonstrated tumor-shrinking effects in patients previously administered irinotecan and with RAS mutants.

This study had certain limitations. First, the small number of patients owing to the early onset of DLTs prevented statistical adjustments and exploratory analyses, including KRAS status. Additionally, the distribution of patients at each level was not adequately adjusted. Second, pharmacokinetics could not be performed owing to institutional limitations, but the results of other studies refer to the pharmacokinetics of the three-drug combination [21]. Third, the relative dose intensity of irinotecan was lower than that of other irinotecan-containing regimens [21-23]. Fourth, all cases were UGT1A1 wild-type cases to ensure safety; however, applying it to other cases may be possible. Finally, the registration period was lengthy, with frequent transfers of the person in charge, leading to a 5-year registration of the remaining 10 cases. However, the same regimen was safely administered to four patients, suggesting that this dose may be safe.

## Conclusions

In summary, the dose-escalation/expansion study findings suggest that the triple combination of TAS-102, irinotecan, and bevacizumab is a feasible treatment option for patients with mCRC resistant to standard therapies. Further evaluation of this regimen is warranted. Given the established use of bevacizumab-fluoropyrimidine-irinotecan chemotherapy in first-line mCRC treatment, these results open the possibility of investigating the TAS-102-bevacizumab-irinotecan combination in earlier treatment lines.
